# Comparative Evaluation of Color Stability in Nanohybrid and Microhybrid Composites Exposed to Misswake Mouthwash: An In Vitro Analysis

**DOI:** 10.1155/ijod/7788956

**Published:** 2025-12-02

**Authors:** Parisa Bazin, Shadab Safarzadeh Khosroshahi, Saeedeh Ebrahimgol, Farnaz Mahdisiar

**Affiliations:** ^1^Doctor of Dental Surgery, Independent General Dentist, Tehran, Iran; ^2^Department of Operative Dentistry, Faculty of Dentistry, Tehran Medical Sciences, Islamic Azad University, Tehran, Iran; ^3^Operative and Aesthetic Dentistry Resident, Faculty of Dentistry, Tehran Medical Sciences, Islamic Azad University, Tehran, Iran

**Keywords:** color stability, mouthwash, nanohybrid and microhybrid composites

## Abstract

**Objective:**

This study aimed to evaluate the color stability of various restorative materials after immersion in one commercial mouthwash.

**Materials and Methods:**

Twenty-four samples from two composite resin systems (Denfil and Voco) and their flowable counterparts (Denfil Flow and Voco Grandio Flow) were exposed to Misswake Total Care mouthwash. Color measurements (*L*^*∗*^, *a*^*∗*^, *b*^*∗*^) were taken before and after immersion using a spectrophotometer, and color differences were calculated according to CIE76 (Δ*E*76) and CIEDE2000 (Δ*E*_00_) formula. Data were analyzed using two-way ANOVA (*α* = 0.05).

**Results:**

All composite groups exhibited color changes after immersion in the mouthwash. Denfil Flow showed the highest mean Δ*E*_00_ (1.11 ± 0.29), followed by Denfil (1.09 ± 0.33), Voco Grandio Flow (0.97 ± 0.20), and Voco Grandio (0.66 ± 0.17). A significant difference was observed between Denfil and Voco groups (*p*  < 0.05), whereas flowable and packable types within the same brand did not differ significantly. All Δ*E* values remained below the clinically perceptible threshold (Δ*E* < 3.3).

**Conclusion:**

Nanohybrid composites demonstrated greater resistance to discoloration than microhybrid composites. Although Misswake Total Care mouthwash induced measurable color changes, these changes remained within clinically acceptable limits. The immersion period used in this study corresponds to approximately 2 years of daily use, suggesting that the product is unlikely to cause clinically relevant discoloration over this timeframe.

## 1. Introduction

The increasing public emphasis on esthetics has led to a rising demand for tooth-colored restorative materials that closely replicate natural teeth [1, 2]. This demand has driven advancements in esthetic dentistry, particularly in the development of new restorative resins. Modern universal composite systems combine the properties of earlier hybrid and microfilled composites, while improvements in filler technology have enhanced both mechanical properties and esthetic performance. One notable innovation in recent years has been the application of nanotechnology to resin composites [3].

Various types of composite resins, including condensable/packable, flowable, microhybrid, and nanocomposite materials, have been introduced to meet essential requirements such as light-curability, accurate color matching, and long-term stability [4, 5]. Despite these advancements, color stability remains a challenge, influenced by internal factors (changes in fillers and matrix) and external factors, including stain absorption, ultraviolet light, thermal and moisture variations, saliva, food, beverages, and mouthwashes [4, 6, 7].

Mouthwashes are increasingly used for antimicrobial control; however, some components, such as chlorhexidine (CHX), can cause color changes in resin composites [8]. The primary aim of antimicrobial mouth rinses is to reduce dental plaque and control the development of periodontal diseases and dental caries [9].

Misswake Total Care, a CPC-based mouthwash containing sodium fluoride, has recently entered the market. It incorporates the antimicrobial agent CPC within a highly bioactive matrix [10]. CPC is a reliable and safe quaternary ammonium compound suitable for therapeutic use [11]. Importantly, this mouthwash does not contain CHX, which can compromise the color stability of resin composite restorations, making it suitable for daily use [12]. Sodium fluoride, a potent anti-caries agent, is also included and may influence color stability [13].

To our knowledge, no studies have evaluated the effect of CPC-based Misswake mouthwash on the color stability of both nanohybrid and microhybrid resin composites. Therefore, this study aimed to determine its effect on the color stability of GrandioVoco, Grandio Flow Voco, Denfil, and Flow Denfil composites. Color evaluation was performed using the CIE color system (*L*^*∗*^, *a*^*∗*^, *b*^*∗*^), with a clinically accepted color change threshold of Δ*E*  < 3.3 [14, 15].

## 2. Materials and Methods

This experimental in vitro study evaluated the color stability of four composite resins after immersion in a commercially available mouthwash. The rationale for selecting only one mouthwash (Misswake Total Care) was based on its widespread clinical use in Iran and the presence of both fluoride and cetylpyridinium chloride as active ingredients, which are relevant to daily oral hygiene practices. However, the absence of comparison groups is acknowledged as a limitation for broader generalization.

### 2.1. Composite Resins

Four composite resins were tested, differing in filler type, particle size, and resin matrix composition (Table 1).

• Voco Grandio A1 and BW are nanohybrid composites with nanofiller particles (barium-aluminosilicate + fumed silica, 20–40 nm).
• Denfil and Denfil Flow are microhybrid composites with microsized filler particles (barium-aluminosilicate + fumed silica, 0.5–1.0 µm).

### 2.2. Mouthrinse

The tested mouthrinse was Misswake Total Care (Seylaneh Sabz, Iran). Its composition is summarized in Table 2.

### 2.3. Sample Preparation

**Figure 1:**
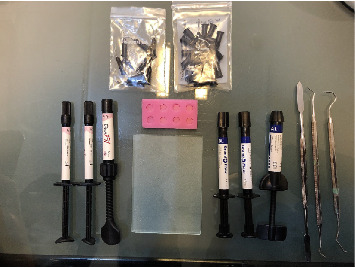
Materials and tools used in this research.

**Figure 2:**
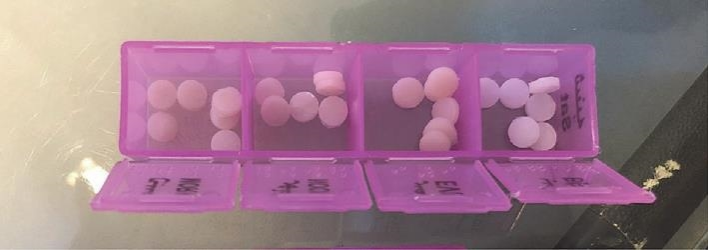
Prepared samples.

**Figure 3:**
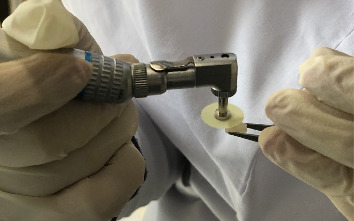
The samples being polished.

Composite specimens were prepared using cylindrical plastic molds (7 mm diameter and 2 mm thickness). The material was condensed and covered on both sides with a 1 mm glass slide to minimize air entrapment and surface irregularities. Polymerization was carried out with an LED curing unit (Bluephase C8, Ivoclar Vivadent, 800 mW/cm^2^) for 40 s. The curing light intensity was verified before each use with a radiometer. Specimens were finished with Bisco finishing disks (medium and fine, 20 s each). Figures 1–3 show the materials, tools, and prepared specimens.

### 2.4. Color Measurement

Specimens were stored in distilled water at 37°C for 24 h for postcuring. Baseline color was measured using a spectrophotometer (CS-2000, Konica Minolta, Japan) on a standard white background, under D65 illumination and a 2° observer, following CIE recommendations. The spectrophotometer probe was positioned perpendicular to the specimen surface at a fixed distance of 10 mm to ensure standardized color measurements. The color parameters (*L*^*∗*^, *a*^*∗*^, *b*^*∗*^) were recorded three times per specimen, and the mean value was used.

### 2.5. Immersion Protocol

Specimens were immersed in Misswake mouthwash for 24 h at 37°C, corresponding to 2 minutes of daily mouthrinse use over 2 years. The use of a static immersion protocol instead of cyclic immersion is acknowledged as a methodological limitation, as it simplifies but does not fully replicate intraoral conditions. After immersion, specimens were rinsed under running water for 120 s and reevaluated for color parameters.

### 2.6. Color Difference (Δ*E*) Calculation

Color changes were quantified using two formulas: the classical CIELAB Δ*E*^⁣^*∗*^^*ab*(Δ*E*76) and the more perceptually uniform CIEDE2000 (Δ*E*_00_). The Δ*E*_00_ was calculated as follows:

(1) $$\begin{array}{c} \Delta E76=\left[{\left(\Delta L\right)}^{2}+{\left(\Delta a\right)}^{2}+{\left(\Delta b\right)}^{2}\right], \end{array}$$

(2) $$\begin{array}{c} \Delta E_{00}^{*}=\sqrt{{\left(\frac{\Delta L^{\prime }}{k_{\text{L}}S_{\text{L}}}\right)}^{2}+{\left(\frac{\Delta C^{\prime }}{k_{\text{C}}S_{C}}\right)}^{2}+{\left(\frac{\Delta H^{\prime }}{k_{\text{H}}S_{\text{H}}}\right)}^{2}+R_{T}\frac{\Delta C^{\prime }}{k_{\text{C}}S_{\text{C}}}\frac{\Delta H^{\prime }}{k_{\text{H}}S_{\text{H}}}}, \end{array}$$

where Δ*L*′, Δ*C*′, and Δ*H*′ represent the differences in lightness, chroma, and hue between two color measurements; *S*_L_, *S*_C_, and *S*_H_ are the weighting functions; *k*_L_, *k*_C_, and *k*_H_ are parametric factors set to 1; and *R*_T_ is the rotation function accounting for the interaction between chroma and hue differences.

Both Δ*E*76 and Δ*E*_00_ values were calculated for all specimens to allow comparison with previous studies and to ensure clinical relevance.

Repeatability of measurements was assessed by remeasuring 10 randomly selected specimens 1 week later by the same operator, yielding an intraclass correlation coefficient (ICC) > 0.90, indicating high repeatability.

### 2.7. Statistical Analysis

The data (*L*^*∗*^, *a*^*∗*^, *b*^*∗*^ values, Δ*E*76 and Δ*E*_00_) were analyzed using two-way ANOVA in PASS 15 software (NCSS, USA), with significance set at *α* = 0.05.

## 3. Results

A two-way ANOVA statistical analysis was conducted to investigate the effects of composite brand (Voco, Denfil), type (packable, flow), and their interaction on the color change after exposure to Misswake Total Care mouthwash.

**Figure 4:**
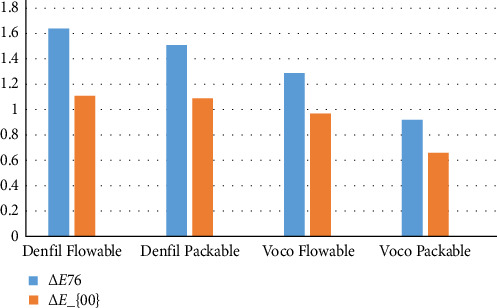
The bar chart showing mean color changes of composite resins after mouthwash exposure.

Table 3 presents the descriptive statistics of the results. The mean Δ*E*_00_ values demonstrated that Voco Grandio (packable) exhibited the lowest color change (Δ*E* = 0.66), representing the best color stability, while Denfil Flow exhibited the highest color change (Δ*E* = 1.11), indicating the poorest stability. The Denfil packable (Δ*E* = 1.09) and Voco Grandio Flow (Δ*E* = 0.97) groups showed intermediate values (Figure 4). Both Δ*E*76 (classical CIELAB) and Δ*E*2000 values were calculated to provide a more perceptually accurate assessment of color changes. Mean Δ*E*2000 values were slightly lower than Δ*E*76, indicating perceptual corrections for human vision.

Lab parameter analysis revealed that, except for the Voco Grandio group, all composites became darker following immersion. In the Voco Grandio group, the most prominent change occurred in the b^*⁣*^*∗*^^ parameter, indicating a shift toward yellowing (Table 4).

According to the two-way ANOVA test (Table 5), significant differences in color change were observed between the Voco Grandio and Denfil groups (*p*  < 0.05), while no significant differences were found between the flowable and packable counterparts within each brand (*p* > 0.05).

## 4. Discussion

Resin composite discoloration can occur over time due to internal or external factors. Internal discoloration may result from aging of composite components such as residual monomers and the activator–initiator system [16, 17]. External discoloration is often caused by food, beverages, and mouthwashes. While mouthwashes offer antimicrobial and anti-inflammatory benefits, frequent use may contribute to staining [18].

The susceptibility of composites to extrinsic staining is influenced by filler particle size, distribution, and resin matrix composition [14, 19]. In this study, Voco composites contained nanosized fillers (barium-aluminosilicate + Fumed silica, 20–40 nm), whereas Denfil composites contained microsized fillers (0.5–1.0 µm). Flowable and packable types differed mainly in filler loading and viscosity, while filler type and particle size were the same.

Previous studies suggest that smaller filler particles improve polishability and surface smoothness, which may reduce susceptibility to discoloration [20, 21]. Similarly, the resin matrix affects water sorption and staining; hydrophilic monomers like Bis-GMA and TEGDMA increase water uptake, while hydrophobic monomers such as UDMA and Bis-EMA reduce it, influencing stain resistance [22].

Color changes were measured instrumentally using a spectrophotometer, which provides reliable Δ*E* values by comparing baseline and postimmersion measurements [23]. In this study, Voco composites exhibited greater color stability than Denfil composites after immersion in Misswake Total Care mouthwash. All Δ*E* values remained below the clinically perceptible threshold (Δ*E*  < 3.3).These findings are consistent with Al-Shami et al. [24], who reported that nanohybrid composites were more color-stable than microhybrid composites under exposure to staining solutions. It is important to note that this study only compares the actual filler size and type differences; conclusions about filler content or cross-linking cannot be drawn due to lack of specific data.

Previous studies have also indicated that composite color stability depends on material characteristics such as filler type and resin matrix composition [3, 25, 26].

Previous studies have also shown that various mouthrinses can affect the color stability of composites, with the fluoride content being one factor contributing to these effects [27, 28]. Alcohol and low pH in some solutions may compromise surface integrity, facilitating staining [9, 29]. In the present study, immersion in Misswake Total Care mouthwash led to statistically significant color changes compared to distilled water, though the changes were not visually perceptible and remained within clinically acceptable limits (Δ*E*  < 3.3).

Our results partially contrast with those of ElEmbaby (1), who reported clinically unacceptable color changes (Δ*E* > 3.3) in some resin composites after immersion in different mouthwashes. Differences in resin formulation and study design may explain this discrepancy. Meshki et al. [30] reported that microhybrid composites demonstrated slightly higher color stability than nanohybrid composites under certain finishing conditions; however, in our study, the nanohybrid Voco composites maintained superior color stability in the context of mouthwash immersion.

Overall, the findings indicate that both filler type and resin matrix composition influence color stability. While all tested composites underwent measurable color changes after immersion, the changes remained clinically acceptable. Future studies are recommended to assess the effects of other mouthwashes, longer immersion periods, and additional intraoral factors such as saliva exposure and brushing, to better replicate real-life conditions and predict long-term color stability.

## 5. Conclusion

All samples showed color changes after immersion, with statistically significant differences observed between the restorative materials and mouthwashes (*p*  < 0.05). The Denfil group exhibited a greater degree of color change compared to the Voco group. However, no significant difference in color change was found between Voco Grandio Flow and Voco Grandio or between Denfil Flow and Denfil when exposed to Misswake Total Care mouthwash. Importantly, none of the samples exceeded the clinically detectable threshold and were deemed clinically acceptable (Δ*E*  < 3.3).

Future studies are recommended to investigate the effects of other mouthwashes on the color stability of common esthetic composites and to explore the impact of varying immersion times on color changes. To better simulate real-life conditions, it is suggested that future research include factors such as saliva exposure and brushing to closely replicate the oral environment, as these play a significant role in the long-term color stability of resin composites.

## Figures and Tables

**Table 1:** Types of composite resins.

Composite types	Shade	Country	Filler type/size	Resin matrix composition	Batch number
Voco Grandio	A1	Germany	Barium aluminosilicate Fumed silica (20–40 nm)	Bis-GMA, TEGDMA	1,918,383
Voco Grandio	BW	Germany	Barium aluminosilicate Fumed silica (20–40 nm)	Bis-GMA, TEGDMA, HEDMA	19,191,209
Denfil Flow	A1	Korea	Barium aluminosilicate Fumed silica (0.05–1 μm)	Bis-GMA TEGDMA, others	DF0304A1
Denfil	A1	Korea	Barium aluminosilicate Fumed silica (0.05–1 μm)	Bis-GMA, TEGDMA, others	DF0304A1

**Table 2:** Characteristics of the mouthwash.

Mouthrinse	Ingredients	Active ingredients	Alcohol	pH	Coloring agents
Misswake total care	Aqua, alcohol, benzoic acid, flavor, poloxamer 407, sorbitol, sodium saccharin, propylene glycol, sodium benzoate	Sodium fluoride (230 ppm F, 0.05%) cetylpyridinium chloride (0.07%)	11%	6.5	None

**Table 3:** Descriptive statistics of color changes.

Descriptive statistics of color changes (Δ*E*76 and Δ*E*_00_)										
Group		*N*	Minimum Δ*E*76	Maximum Δ*E*76	Mean Δ*E*76	SD Δ*E*76	Minimum Δ*E*_00_	Maximum Δ*E*_00_	Mean Δ*E*_00_	SD Δ*E*_00_
Denfil	Packable	6	0.94	2.29	1.51	0.50	0.67	1.60	1.09	0.33
	Flow	6	1.11	2.15	1.64	0.39	0.77	1.48	1.11	0.29

Voco	Packable	6	0.69	1.23	0.92	0.22	0.46	0.91	0.66	0.17
	Flow	6	1.09	1.45	1.29	0.15	0.78	1.33	0.97	0.20

**Table 4:** Descriptive statistics by group.

Group			Minimum	Maximum	Mean	Standard deviation
Denfil	Packable (*n* = 6)	Δ*L*	−2.04	0.88	−0.6383	1.20558
		Δ*a*	−0.63	0.19	−0.0517	0.31192
		Δ*b*	−0.63	1.40	0.6183	0.70511
		Δ*E*76	0.94	2.29	1.5134	0.50030
	Flow (*n* = 6)	Δ*L*	−1.97	−0.88	−1.2700	0.44027
		Δ*a*	0.08	0.38	0.1950	0.09955
		Δ*b*	0.64	1.31	0.9817	0.24277
		Δ*E*76	1.11	2.15	1.6444	0.39430

Voco	Packable (*n* = 6)	Δ*L*	−1.20	1.07	0.0533	0.85066
		Δ*a*	−0.02	0.64	0.2517	0.21693
		Δ*b*	−0.42	0.84	0.2767	0.45289
		Δ*E*76	0.69	1.23	0.9585	0.21131
	Flow (*n* = 6)	Δ*L*	−1.44	−0.91	−1.1533	0.19284
		Δ*a*	0.13	0.79	0.2883	0.24903
		Δ*b*	0.05	0.59	0.3867	0.20858
		Δ*E*76	1.09	1.45	1.2892	0.15389

**Table 5:** Tests of between-subject effects.

Tests of between-subjects effect					
Dependent variable:	Δ*E*76				
Source	Type III sum of squares	Df	Mean square	*F*	Sig.
Corrected model	1.622	3	0.541	4.562	0.014
Intercept	43.829	1	43.829	369.778	0.000
Group	1.242	1	1.242	10.483	0.004
Mode	0.320	1	0.320	2.697	0.116
Group *×* mode	0.060	1	0.060	0.505	0.485
Error	2.371	20	0.119	—	—
Total	47.821	24	—	—	—
Corrected total	3.993	23	—	—	—

## Data Availability

The data supporting the findings of this study are available from the corresponding author upon reasonable request.

## References

[1] ElEmbaby A. E. (2014). The Effects of Mouth Rinses on the Color Stability of Resin-Based Restorative Materials. *Journal of Esthetic and Restorative Dentistry*.

[2] Al-Samadani K. (2017). The Effect of Preventive Agents (Mouthwashes/Gels) on the Color Stability of Dental Resin-Based Composite Materials. *Dentistry Journal*.

[3] Reddy P. S., Tejaswi K. S., Shetty S., Annapoorna B., Pujari S. C., Thippeswamy H. (2013). Effects of Commonly Consumed Beverages on Surface Roughness and Color Stability of the Nano, Microhybrid and Hybrid Composite Resins: An In Vitro Study. *The Journal of Contemporary Dental Practice*.

[4] Khosravi M., Esmaeili B., Nikzad F., Khafri S. (2016). Color Stability of Nanofilled and Microhybrid Resin-Based Composites Following Exposure to Chlorhexidine Mouthrinses: An In Vitro Study. *Journal of Dentistry*.

[5] Puckett A. D., Fitchie J. G., Kirk P. C., Gamblin J. (2007). Direct Composite Restorative Materials. *Dental Clinics of North America*.

[6] Harorlı O. T., Barutcigil Ç. (2014). Color Recovery Effect of Commercial Mouth Rinses on a Discolored Composite. *Journal of Esthetic and Restorative Dentistry*.

[7] Jyothi K., Crasta S., Venugopal P. (2012). Effect of Five Commercial Mouth Rinses on the Microhardness of a Nanofilled Resin Composite Restorative Material: An In Vitro Study. *Journal of Conservative Dentistry*.

[8] Tadakamadla S. K., Bharathwaj V. V., Duraiswamy P., Sforza C., Tartaglia G. M. (2020). Clinical Efficacy of a New Cetylpyridinium Chloride-Hyaluronic Acid–Based Mouthrinse Compared to Chlorhexidine and Placebo Mouthrinses—A 21-Day Randomized Clinical Trial. *International Journal of Dental Hygiene*.

[9] Celik C., Yuzugullu B., Erkut S., Yamanel K. (2019). Effects of Mouth Rinses on Color Stability of Resin Composites. *European Journal of Dentistry*.

[10] Hu D., Li X., Sreenivasan P. K., DeVizio W. (2009). A Randomized, Double-Blind Clinical Study to Assess the Antimicrobial Effects of a Cetylpyridinium Chloride Mouth Rinse on Dental Plaque Bacteria. *Clinical Therapeutics*.

[11] Hazar A., Hazar E. (2024). Effects of Different Antiviral Mouthwashes on the Surface Roughness, Hardness, and Color Stability of Composite CAD/CAM Materials. *Journal of Applied Biomaterials and Functional Materials*.

[12] Allccahuaman-Avalos R., Medina-Sánchez R., Castro-Ramirez L. (2023). In Vitro Color Stability Evaluation of Three Polished and Unpolished Nanohybrid Resin Composites Immersed in a 0.12% Chlorhexidine-Based Mouthwash at Different Times. *Polymers*.

[13] Sharma A., Agarwal N., Anand A., Jabin Z. (2018). To Compare the Effectiveness of Different Mouthrinses on *Streptococcus mutans* Count in Caries Active Children. *Journal of Oral Biology Craniofacial Research*.

[14] Festuccia M. S. C. C., Garcia L. D. F. R., Cruvinel D. R., Pires-De-Souza F. D. C. P. (2012). Color Stability, Surface Roughness and Microhardness of Composites Submitted to Mouthrinsing Action. *Journal of Applied Oral Science*.

[15] Liu S. M. (2004). The Development of a Portable Spectrophotometer for Noncontact Color Measurement. *IEEE Transaction on Instrumentation and Measurement*.

[16] Hamdy T. M., Abdelnabi A., Othman M. S., Bayoumi R. E., Abdelraouf R. M. (2023). Effect of Different Mouthwashes on the Surface Microhardness and Color Stability of Dental Nanohybrid Resin Composite. *Polymers*.

[17] El-Rashidy A. A., Abdelraouf R. M., Habib N. A. (2022). Effect of Two Artificial Aging Protocols on Color and Gloss of Single-Shade Versus Multi-Shade Resin Composites. *BMC Oral Health*.

[18] Kepler L. C., Rodrigues A. P. M., Dall Agnol M. A., Rodrigues-Junior S. A. (2021). Effect of Whitening Mouth Rinses on the Chemical and Physical Properties of a Nanofilled Composite. *Brazilian Journal of Oral Sciences*.

[19] Rodrigues S. A., Scherrer S. S., Ferracane J. L., Bona Á. D. (2008). Microstructural Characterization and Fracture Behavior of a Microhybrid and a Nanofill Composite. *Dental Materials*.

[20] Ajay R., Kumar M. S., Miskeen Sahib S. (2017). Color Stability Assessment of Two Different Composite Resins With Variable Immersion Time Using Various Beverages: An In Vitro Study. *Journal of Pharmacy and Bioallied Sciences*.

[21] Kalita T., Kalita C., Das L. (2023). Comparative Evaluation of Colour Stability and Surface Roughness of Nanohybrid Composite Resins in Mouth Rinse and Colouring Beverages. *Cureus*.

[22] Ertas E., Güler A. U., Yücel A. Ç., Köprülü H. C., Güler E. (2006). Color Stability of Resin Composites After Immersion in Different Drinks. *Dental Materials Journal*.

[23] Johnston W. M. (2009). Color Measurement in Dentistry. *Journal of Dentistry*.

[24] Al-Shami A. M., Alshami M. A., Al-Kholani A. I., Al-Sayaghi A. A. M. (2023). Color Stability of Nanohybrid and Microhybrid Composites After Immersion in Common Coloring Beverages at Different Times: A Laboratory Study. *BDJ Open*.

[25] Sunbul H. A., Silikas N., Watts D. C. (2016). Surface and Bulk Properties of Dental Resin- Composites After Solvent Storage. *Dental Materials*.

[26] Ribeiro J. S., Peralta S. L., Salgado V. E., Lund R. G. (2017). In Situ Evaluation of Color Stability and Hardness’ Decrease of Resin-Based Composites. *Journal of Esthetic and Restorative Dentistry*.

[27] Ahuja P., Ahuja V., Jindal N., Aggarwal R. (2022). Comparative Evaluation of Effect of Different Mouthwashes on Color Stability of Different Composites – An In Vitro Study. *University Journal of Dental Sciences*.

[28] Diab M., Zaazou M., Mubarak E., Olaa M. (2007). Effect of Five Commercial Mouthrinses on the Microhardness and Color Stability of Two Resin Composite Restorative Materials. *Australian Journal of Basic Applied Sciences*.

[29] Moreira A. D., Mattos C. T., de Araújo M. V. A., Ruellas A. C. D. O., Sant’Anna E. F. (2013). Chromatic Analysis of Teeth Exposed to Different Mouthrinses. *Journal of Dentistry*.

[30] Meshki R., Rashidi M. (2022). Effect of Natural and Commercially Produced Juices on Colour Stability of Microhybrid and Nanohybrid Composites. *BDJ Open*.

